# Diversity and Molecular Characterization of Mosquitoes (Diptera: Culicidae) in selected ecological regions in Kenya.

**DOI:** 10.12688/f1000research.18262.1

**Published:** 2019-03-06

**Authors:** Moni Makanda, Gladys Kemunto, Lucy Wamuyu, Joel Bargul, Jackson Muema, James Mutunga

**Affiliations:** 1 Institute for Basic Sciences Technology and Innovation, Pan African University, Nairobi, P.O. Box 62000-00200, Kenya; 2 Zoology Department, Jomo Kenyatta University of Agriculture and Technology, Nairobi, P.O. Box 62000-00200, Kenya; 3 Institute of Biotechnology Research, Jomo Kenyatta University of Agriculture and Technology, Nairobi, P.O. Box 62000-00200, Kenya; 4 Biochemistry Department, Jomo Kenyatta University of Agriculture and Technology, Nairobi, P.O. Box 62000-00200, Kenya; 5 Biological Sciences Department, Mount Kenya University, Thika, P.O. Box 342-01000, Kenya

**Keywords:** Aedes, Anopheles, Culex, chikungunya, Rift valley fever, dengue fever

## Abstract

Mosquitoes play a predominant role as leading agents in the spread of vector-borne diseases and consequent mortality in humans. Despite reports on increase of new and recurrent mosquito borne-disease outbreaks such as chikungunya, dengue fever and Rift valley fever in Kenya little is known about the genetic characteristics and diversity of the vector species that have been incriminated in transmission of disease pathogens. In this study, we identified mosquito species across Kisumu, Kilifi and Nairobi Counties and determined their genetic diversity and phylogenetic relationships. PCR was used to amplify and sequence the partial cytochrome oxidase subunit 1 (CO1) gene of mosquito samples. Molecular-genetic and phylogenetic analysis of the partial cytochrome oxidase subunit 1 (CO1) gene was employed to identify their relationships with known mosquito species. Fourteen (14) haplotypes belonging to genus *Aedes*, nine (9) haplotypes belonging to genus *Anopheles* and twelve (12) haplotypes belonging to genus *Culex *were identified in this study. Findings from this study revealed a potentially new haplotype belonging to *Anopheles* genus and reported the first molecular characterization of *Aedes cummnisii* in Kenya. Sequence results revealed variation in mosquito species from Kilifi, Kisumu and Nairobi. Since vector competence varies greatly across species and species-complexes and is strongly associated with specific behavioural adaptations, proper species identification is important for vector control programs.

## Introduction

Mosquitoes are vectors responsible for transmission of numerous pathogens causing diseases such as; malaria, lymphatic filariases, avian malaria and arboviruses such as Dengue virus, Chikungunya virus, Yellow fever, West Nile Fever, and Zika virus ( Charrel *et al*., 2007; Semenza, 2014). Africa is one of the major hosts of mosquitoes responsible for mosquito-borne viruses ( Braack *et al*., 2018) that are of great medical importance and contribute to the current global public health threat ( Enserink, 2007; Gubler, 2002; Higgs, 2014). Seasonal and environmental changes play a role in the global distribution of mosquito species and the arboviruses they transmit ( Anyamba *et al*., 2001; Hasnan *et al*., 2016). The global spread of vector-borne diseases has resulted to multiple calls on nations to enhance surveillance of emerging arboviruses that requires understanding of the species composition and distribution of potential mosquito vectors ( Grout *et al*., 2017; Kollars *et al*., 2016).

In the recent past, there has been an increasing spread of mosquito-borne viruses such as Chikungunya, dengue fever and Rift Valley fever in Kenya, thus prompting a need for further research ( Johnson *et al*., 1982; Konongoi *et al*., 2018). The available literature on mosquitoes in Kenya mainly addresses aspects of morphological identification of mosquito vectors and limited molecular characterization ( Lutomiah *et al*., 2013; Mwangangi *et al*., 2013). Despite mosquitoes being a major public health challenge in Kenya, little is known about their species diversity and distribution along different ecological zones such as the Kenyan coast and Kenya’s capital city. Subsequently, population genetic studies on mosquito vectors in Kenya have focused on the *Anopheles* genus because of its importance in endemic malaria transmission ( Chen *et al*., 2006; Chen *et al*., 2004; Lukindu *et al*., 2018). In addition, most of the studies on mosquito vector composition and diversity are based on mosquitoes confined to a single habitat or with a limited habitat range ( Ajamma *et al*., 2016a; Muturi *et al*., 2006). The species composition and distribution of *Anopheline* mosquitoes in Kenya, particularly along the Kenyan coast, have broadly been reported over that of *Culcine* mosquitoes ( Mbogo *et al*., 2003; Midega *et al*., 2007, Midega *et al*., 2010). Moreover, little has been documented on the species composition and diversity of all mosquito groups by use of molecular markers. As such, understanding the species composition and diversity patterns of the suggested vectors is pivotal to the judicious deployment of existing vector control strategies and the development of new effective vector control interventions ( Kraemer *et al*., 2016).

In this study, we employed molecular genetic techniques, involving PCR and sequencing of cytochrome oxidase subunit 1 (CO1) gene to identify and characterize mosquito species in Nairobi, Kisumu and Kilifi Counties in Kenya.

## Methods

### Study sites

**Figure 1.**
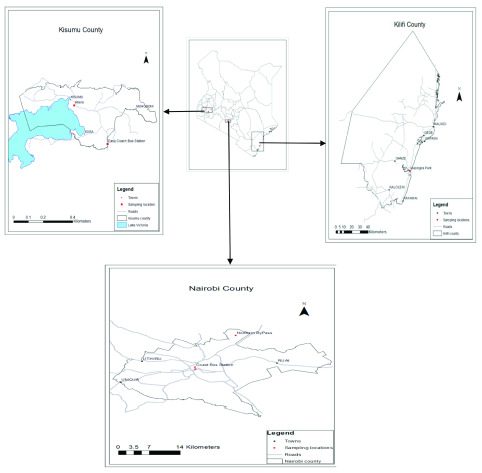
A map of Kenya showing the location of sampled mosquitoes.

This study was carried out at Nairobi, Kilifi and Kisumu Counties in Kenya. Kilifi and Kisumu regions were chosen purposively due to their high abundance of mosquito vectors ( WHO, 2017) and vector-borne disease burden, while Nairobi region was selected because it’s a major international and domestic destination for both humans and parasites ( Wesolowski *et al*., 2012). Two sampling sites were randomly selected from each of the three regions as follows: Kisumu; Ahero and Kisumu town, Kilifi; Kilifi town and Mazingira Park and Nairobi; Nairobi city centre, and Northern Bypass ( Figure 1).

### Sampling strategy

The trapping of mosquitoes was carried out in the respective counties during the dry season (January–February 2018) and wet season (March–April 2018). The captures were conducted day and night using the Pyrethrum Spray Catch (PSC) method ( Ndiath *et al*., 2011). The specimens were adult mosquitoes, which were morphologically sorted in the field into their respective genera, and transported in liquid nitrogen to the laboratory for further molecular analysis. A total of 2,438 adult mosquitoes were collected. Of these, 894, 824 and 720 adult mosquito samples were collected in Nairobi, Kisumu and Kilifi respectively. From the overall collection, 300 hundred mosquitoes per county were randomly selected for PCR. A total of 25 sequences per study region were used for phylogenetic and genetic diversity analysis ( Hale *et al*., 2012).

### PCR analysis

Total genomic DNA was extracted from whole body of individual mosquitoes using the Collins’ protocol ( Collins *et al*., 1987) with minor modifications. A DNA homogenizing buffer (containing 0.1 M NaCl, 0.2 M sucrose, 0.01 M EDTA and 0.03 M Tris pH 8) was mixed with a lysis buffer (containing 0.25 M EDTA, 2.5% w/v SDS and 0.5 M Tris pH 9.2) in the ratio 4:1 to make up the grinding buffer (GB). Each mosquito was homogenized in 100 цl of the GB, using a hand-held pestle homogenizer and incubated for 30 min at 55°C. Into each sample, 14 цl of 8 M potassium acetate (KAc), a deproteinating reagent was added and then incubated for 30 min at room temperature before centrifuging at 13,000 rpm for 15 min to get the supernatant that contains the nucleic acid component. Ethanol precipitation 95% was used to precipitate the genomic DNA. Centrifugation at 13,000 rpm for 10 minutes to obtain a pellet that is the nucleic acid. Followed by a washing step using 70% ethanol. The DNA pellet was suspended in 100 μL of T.E buffer pH 7.2 and stored at –20°C awaiting subsequent experimental procedures.

The primer set ;Forward (LCO1490_GGTCAACAAATCATAAAGATATTGG) and Reverse (HCO2198 TAAACTTCAGGGTGACCAAAAAATCA) synthesized by Macrogen (OG180803-187) and previously published by Folmer *et al*. were used in molecular identification of the mosquito species ( Folmer *et al*., 1994). In a 10 µL PCR reaction volume, the PCR mix consisted of 2 µL 1× HOT FIREPol® Eva Green mix (Solis BioDyne, Tartu, Estonia) catalogue number 08-31-00008, 6 µL of nuclease-free water, 0.5 picomoles of each primer and 1 µL of the DNA template. The fragments were amplified using applied biosystems ProFlex SN 297802057 thermocycler under the following cycling parameters; initial denaturation for 15 min at 95°C, followed by 35 cycles of denaturation at 95°C for 30 sec, annealing at 50°C ( *Anopheles, Aedes, Culex*) for 30 sec, and extension at 72°C for 30 sec, and a final extension at 72°C for 7 min. The PCR products from the amplification of the mitochondrial cytochrome c oxidase 1 (CO1) region of the mosquito after purification using QIAquick® gel extraction kit catalogue number 28706, were shipped for sequencing at Macrogen Inc., South Korea.

### Sequence analysis

Resultant mitochondrial cytochrome c oxidase 1 (CO1) sequence chromatograms were edited and visualized using Chromas Lite version 2.6.5. The sequences were deposited in GenBank and accession numbers assigned accordingly. Consensus sequences were aligned using ClustalX version 2 ( Thompson *et al*., 1997), and visualized using Seaview Version 4.7 ( Gouy *et al*., 2010). Unique sequences (haplotypes) were identified using DnaSP version 6 ( Librado & Rozas, 2009). Sequence polymorphisms were identified using DnaSP and visualized using Jalview version 2.10.5 ( Waterhouse *et al*., 2009). DNA sequence divergence was analysed using DnaSP. These unique sequences were compared with reference sequences from other parts of the world, selected to represent the *Aedes*, *Anopheles* and *Culex* genera previously reported and available from GenBank ( Benson *et al*., 2011). Other sequences similar to the study sequences in GenBank obtained using the Blastn algorithm were also included in the analysis. Multiple alignment and comparison of the study sequences and GenBank references were performed using ClustalX. Phylogenetic and molecular evolutionary analyses were conducted using Software for Molecular Evolutionary Genetics ( MEGA7) ( Kumar *et al*., 2016). Phylogenetic trees were constructed using the maximum likelihood (ML) method rooted using *Lutzomyia longipalpis*. The phylogenetic trees were estimated using the best-fit general time-reversible (GTR) model of nucleotide substitution with gamma-distributed rate variation among sites. Bootstrap resampling process (1000 replications) was employed to assess the robustness of individual nodes of phylogeny (only >50% were indicated). The resultant tree was visualized using Dendroscope version 3 ( Huson & Scornavacca, 2012).

## Results

### Phylogenetic Analysis

From each study site 25 CO1 gene amplicons were sequenced for phylogenetic analysis. In total, 14 haplotypes belonging to genera *Aedes*, 9 haplotypes belonging to genera *Anopheles* and 12 haplotypes belonging to genera *Culex* were identified through CO1 sequence analysis; sequences were deposited in GenBank and assigned accession numbers ( Table 1). Sequence analysis revealed a unique *Anopheles* haplotype (GenBank accession number, MK300230) ( Figure 3). Subsequently, haplotypes of *Anopheles gambiae, Anopheles funestus, Aedes cumminsii, Aedes aegypti, Culex pipiens* and *Culex sitiens* were found to be distributed across Kilifi, Kisumu and Nairobi mosquito populations ( Table 1).

**Table 1.** Distribution of *Aede*s, *Anopheles* and *Culex* species across Kisumu, Kilifi and Nairobi.

Region	Sample Size	Species	Number of Haplotypes	Accession Number
Nairobi	25	*Aedes aegypti*	4	MK300226, MK300227, MK300228, MK300229
		*Anopheles gambiae*	1	MK300238
		*Culex pipiens*	3	MK300248, MK300249, MK300250
Kisumu	25	*Anopheles gambiae*	5	MK300233, MK300234,MK300235,MK300236, MK300237
		*Anopheles funestus*	2	MK300231, MK300232
		*Culex pipiens*	2	MK300242, MK300247
Kilifi	25	*Aedes aegypti*	9	MK300216,MK300217,MK300218, MK300219, MK300220, MK300221, MK300222, MK300223, MK300224
		*Aedes cumminsii*	1	MK300225
		*Anopheles species*	1	MK300230
		*Culex pipiens*	3	MK300239, MK300242, MK300246
		*Culex sitiens*	5	MK300240, MK30024, MK300243, MK300244, MK300245

Diversity indices for the three populations, based on sequenced results were calculated as shown in ( Table 2). Average number nucleotide differences (k), nucleotide diversity Pi (π) and haplotype diversity (Hd) varied among the species ( Table 2).

**Table 2.** Genetic diversity indices in the mitochondrial cytochrome oxidase 1 (CO1) sequences of mosquito species from Nairobi, Kisumu and Kilifi.

Region	Species	Hap	S	k	Pi (π)	Hd
Nairobi	*Aedes aegypti*	4	21	11.333	0.0160	1.000
	*Culex pipiens*	3	5	3.333	0.0047	1.000
Kisumu	*Anopheles gambiae*	5	7	3.200	0.0045	1.000
	*Anopheles funestus*	2	1	1.000	0.0236	1.000
	*Culex pipiens*	2	6	6.000	0.0085	1.000
Kilifi	*Aedes aegypti*	9	12	4.222	0.0060	1.000
	*Culex pipiens*	3	51	34.000	0.0480	1.000
	*Culex sitiens*	5	53	22.000	0.0311	1.000

^2^Hap: number of haplotypes; S: number of polymorphic Segregating Sites; k: The average number of nucleotide differences; Pi (π): nucleotide diversity; Hd: haplotype gene diversity.

**Figure 2.**
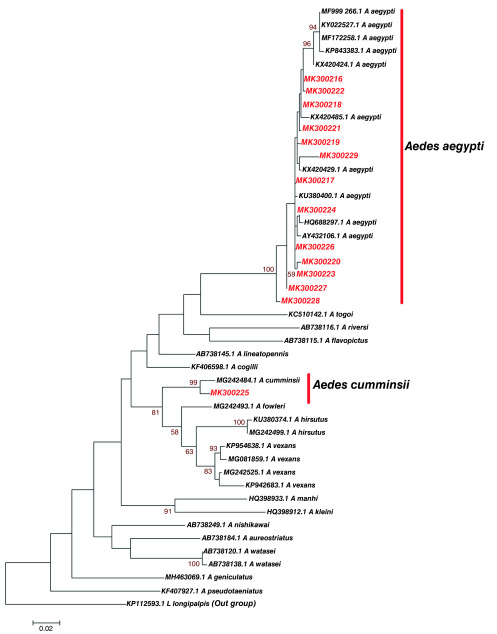
Maximum likelihood phylogenetic tree of partial cytochrome oxidase subunit 1 (CO1) nucleotide sequences of *Aedes* species haplotypes in Red and GenBank references in Black. The scale represents the number of differences between sequences (0.02=2%). The gamma correction for rate heterogeneity was 0.1963. The analysis involved 46 nucleotide sequences. There were a total of 657 positions in the final dataset.

Phylogenetic analysis of fourteen (14) *Aedes* haplotypes from Kilifi and Nairobi with similar sequences based on Blastn (NCBI) search and sequences of known *Aedes* identity revealed that study *Aedes* haplotype Accession number MK300225 clustered with *Aedes cumminsii* ( Figure 2). Study haplotypes Accession number; MK300216, MK300217, MK300218, MK300219, MK300220, MK300221, MK300222, MK300223, MK300224, MK300226, MK300227, MK300228 and MK300229 clustered with *Aedes aegypti* that has been previously identified in France (Accession number HQ688297.1). Significantly, they also clustered with *Aedes aegypti* (Accession number KX420485.1, KX420429.1 and KU380400.1) previously reported in Nyanza-Kisumu, Kenya ( Figure 2). Genetic divergence between study *Aedes* haplotypes identified in Kilifi and Nairobi and *Aedes* species they clustered with (sequences of known species obtained from GenBank) was variable ( Table 3). There was limited divergence between *Aedes aegypti (*Accession number KX420485) that has previously been identified in Nyanza-Kisumu, Kenya and study haplotypes MK300216, MK300222, MK300218 and MK300221. *Aedes aegypti* (Accession number KU380400.1) that has been reported in Nyanza-Kisumu, Kenya before showed limited divergence with study haplotype MK300217. Limited divergence was also identified between haplotype MK300224 and *Aedes aegypti* (Accession number HQ688297.1) that has been characterized in France. Greater divergence and heterogeneity was observed between *Aedes aegypti* and study haplotypes MK300225, MK300219 and MK300229. Study haplotypes MK300216, MK300220, MK300223, MK300227 and MK300228 formed a distinct clade with other *Aedes aegypti* of known identity ( Figure 2).

**Table 3.** Sequence divergence between study *Aedes* species haplotypes and closely associated sequences from GenBank.

	MK 300225	MK 300216	MK 300222	MK 300218	MK 300221	MK 300219	MK 300229	MK 300224	MK 300219
MG242484.1 *Ae.aegypti*	0.017								
KX420485.1 *Ae.aegypti*		0.008	0.009	0.006	0.009	0.013			
KX420429.1 *Ae.aegypti*							0.017		
KU380400.1 *Ae.aegypti*								0.003	
HQ688297.1 *Ae.aegypti*									0.003

**Figure 3.**
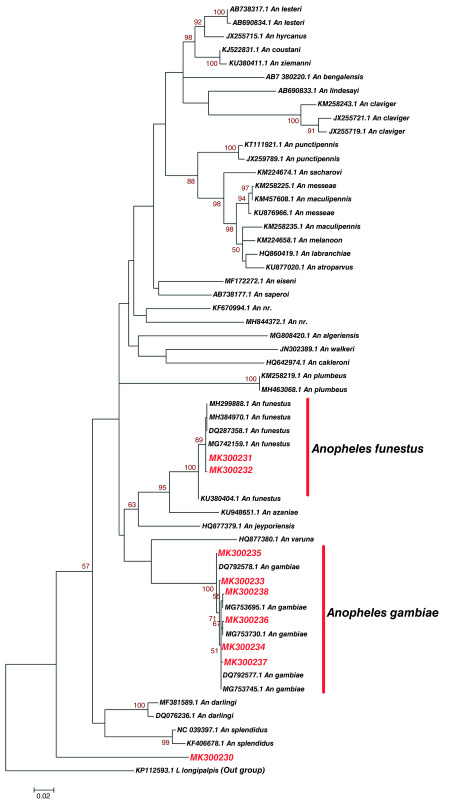
Maximum likelihood phylogenetic tree of partial cytochrome oxidase subunit 1 (CO1) nucleotide sequences of *Anopheles* species haplotypes in Red and GenBank references in Black. The gamma correction for rate heterogeneity was 0.1647. The analysis involved 57 nucleotide sequences. There were a total of 658 positions in the final dataset.

Phylogenetic analysis of haplotypes with similar sequences to those of known identity showed a clustering of study *Anopheles* haplotype MK300231 and MK300232 with *Anopheles funestus.* Notably, they also clustered with *Anopheles funestus* (Accession number MH299888.1 and KU380404.1) that has been reported in Kilifi and Baringo counties in Kenya respectively ( Figure 3). Study haplotype MK300233, MK300234, MK300235, MK300236, MK300237 and MK300238 clustered with *Anopheles gambiae* that have previously been isolated in Uganda (Accession number MG753695.1, MG753730.1 and MG753745.1) ( Figure 3). *Anopheles* haplotype MK300230 formed its own distinct clade. This study haplotype MK300230 may be a new species or novel haplotype not yet described ( Figure 3). Genetic divergence between *Anopheles* haplotypes identified Kisumu, Kilifi, Nairobi and *Anopheles* species they clustered with was variable in some haplotypes while others were not variable ( Table 4). There was very limited divergence and heterogeneity between *Anopheles funestus* and study haplotype MK300231 and MK300232. There was no divergence between *Anopheles gambiae* (Accession number DQ792577.1 and MG753695.1) and study haplotype MK300234. *Anopheles gambiae* (Accession number MG753695.1) has been identified in Uganda before. Study haplotypes MK300235 MK300233 MK300238 MK300236 and MK300237 showed limited divergence with *Anopheles gambiae*.

**Table 4.** Sequence divergence between study *Anopheles* haplotypes and known *Anopheles* species obtained from GenBank.

	MK 300231	MK 300232	MK 300235	MK 300233	MK 300238	MK 300236	MK 300234	MK 300238
MG742159.1 *An.funestus*	0.000	0.002						
MH299888.1 *An.funestus*		0.003						
MH384970.1 *An.funestus*		0.003						
DQ287358.1 *An.funestus*		0.003						
DQ792578.1 *An. gambiae*			0.002					
MG753695.1 *An. gambiae*				0.005	0.002			
MG753730.1 *An. gambiae*						0.002		
DQ792577.1 *An. gambiae*							0.000	0.002
MG753695.1 *An. gambiae*							0.000	0.002

**Figure 4.**
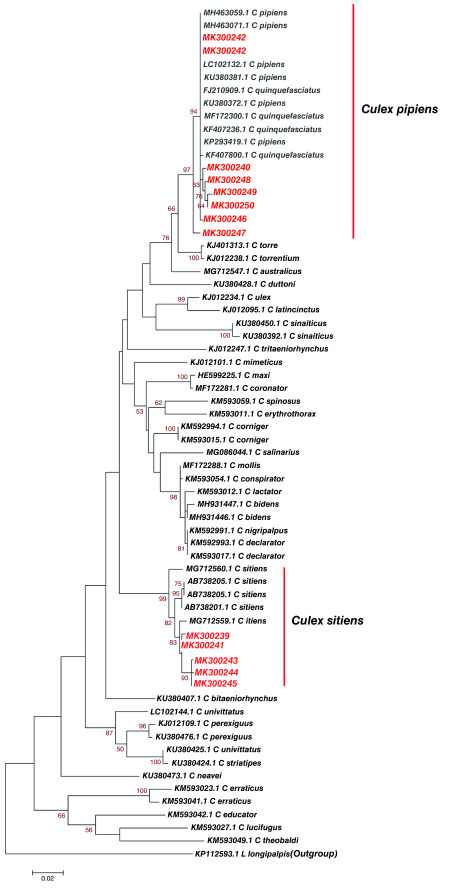
Maximum likelihood phylogenetic tree of partial cytochrome oxidase subunit 1 (CO1) nucleotide sequences of *Culex* species haplotypes in Red and GenBank references in Black. The gamma correction for rate heterogeneity was is 0.1790. The analysis involved 62 nucleotide sequences. There were a total of 658 positions in the final dataset.

From the phylogenetic analysis, we further established that 12 *Culex* haplotypes from Kilifi, Kisumu and Nairobi, and similar sequences of known identity based on Blastn (NCBI) showed a clustering of study haplotype MK300240, MK300242, MK300246, MK300247, MK300248, MK300249 and MK300250 with *Culex pipiens* that have been identified in different regions of the world. Importantly, they clustered with *Culex pipiens* that has previously been identified in Nyanza-Kisumu, Kenya (Accession number KU380381.1, KU380372.1) ( Figure 4). Study haplotypes MK300239, MK300241, MK300243, MK300244, MK300245 clustered with *Culex sitiens* that has been previously identified in Australia (Accession number MG712559.1) ( Figure 4). Genetic divergence between *Culex* haplotypes identified Kisumu, Kilifi, Nairobi and reference *Culex* species was slightly variable in some species, while other species showed no divergence ( Table 5).

**Table 5.** Sequence divergence between study *Culex* species and known *Culex* species obtained from GenBank.

	MK 300242	MK 300246	MK 300239	MK 300241	MK 300243	MK 300244	MK 300245
LC102132.1 *Culex pipiens*	0.000	0.002					
KU380381.1 *Culex pipiens*	0.000	0.002					
KU380372.1 *Culex pipiens*	0.000	0.002					
MG712559.1 *Culex sitiens*			0.009	0.006	0.009	0.001	0.009

## Discussion

This study identified *Aedes aegypti* in both Kilifi and Nairobi populations and *Aedes cummnisii* in the Kilifi population only. *Anopheles gambiae* was identified in both Kisumu and Nairobi population whereas *Anopheles funestus* was identified in Kisumu population only. A potentially novel *Anopheles* haplotype MK300230 was identified in Kilifi population. *Culex pipiens* was identified in all the three populations; Kisumu, Nairobi and Kilifi while *Culex sitiens* was only identified in the Kilifi population. The greatest diversity was in the genus *Aedes* that has 14 haplotypes, followed by *Culex* 12 and *Anopheles* nine, this is consistent with other studies looking at mosquito diversity in different ecological regions in Kenya ( Mwangangi *et al*., 2012). Similarly, out of the 35 mosquitoes haplotypes identified in Kilifi, Nairobi and Kisumu regions, one *Culex* haplotype MK300242 from this study has been previously reported at Kisumu-Nyanza in Kenya and in Portugal ( Ajamma *et al*., 2016b; Mixão *et al*., 2016), and one *Anopheles* haplotype MK300234 in Uganda ( Lukindu *et al*., 2018). The Kilifi mosquito population had the greatest diversity and abundance of mosquito species, possibly due to its geographical position, human activities and natural climatic conditions.

*Aedes cummnisii* has been morphologically identified in Kenya before ( Mwangangi *et al*., 2006), however, this study reports the first molecular characterization of *Aedes cummnisii* in Kenya. *Aedes* haplotypes between Kilifi and Nairobi populations were divergent based on nucleotide diversity tests; this could be due to different climatic zones. Thus, diversity in vector haplotypes plays an important role in vector control and management practices and epidemiology of vector borne diseases ( Murugan *et al*., 2016). Phylogenetic analysis showed presence of two *Aedes* species that is *Aedes cummnisii* and *Aedes aegypti,* in Kilifi, while Nairobi had only *Aedes aegypti* ( Figure 2 and Table 1). This study has identified 4 different *Aedes aegypti* haplotypes in Nairobi. Previous studies have indicated presence of only a few *Aedes aegypti* in Nairobi ( Kinuthia *et al*., 2017). There is therefore an increase in diversity in *Aedes aegypti* species from Nairobi; diversity and spread of *Aedes aegypti* has been associated with expansion on arboviral infection ( Woolhouse *et al*., 1997). The diversity of *Aedes aegypti* in Nairobi could be the result of high population density ( Gubler & Clark, 1995), poor sanitation and waste disposal as well as water management ( Monath, 1994). The Kilifi population had genetically diverse forms of *Aedes aegypti* ( Table 2). *Aedes aegypti* is widespread on the Kenyan coast ( McDonald, 1977; Teesdale, 1955). It is the principal vector of dengue virus, chikungunya, and urban yellow fever virus ( Reiter, 2010), and was predominated in the Kilifi samples. This may contribute to the high susceptibility to dengue-outbreak reported in the region ( Baba *et al*., 2016; Chepkorir *et al*., 2014). Secondly, factors relating to availability of breeding sites, temperature or altitudinal differences may have influenced the diversity patterns of *Aedes aegypti* in Kilifi ( Barrera *et al*., 2011). Evidence of high diversity of *Aedes aegypti* in Kilifi also means that the Kenyan coast is consistently at higher risk of Yellow Fever transmission ( Agha *et al*., 2017). Kilifi lies in between Malindi and Mombasa cities which are popular destinations for international tourism as well as maritime industry, and where *Aedes aegypti* is widespread ( Ngugi *et al*., 2017). Human trade and travel may bolster movement of *Aedes aegypti* ( Powell & Tabachnick, 2013) and contribute to diversity of the species, in addition invasion risk related to human travel has become far more severe ( Egizi *et al*., 2016; Wilder-Smith & Gubler, 2008). Phylogenetic relationship between *Aedes* species from this study and other *Aedes* species of known identity from GenBank showed clustering with *Aedes cummnisii* and *Aedes aegypti* at a high bootstrap value (>90%) at the defining node on the phylogenetic tree ( Figure 2). However, genetic diversity between *Aedes* species from this study and those of known identity from GenBank was variable ( Table 3).

*Anopheles* species were distributed across the three study populations Kisumu, Nairobi and Kilifi ( Table 1). *Anopheles* species between Kilifi, Kisumu and Nairobi populations were highly divergent as analyzed using molecular markers. Nairobi had only one haplotype of *Anopheles gambiae* ( Table 1). *Anopheles* mosquitoes have also been reported in places where malaria has been eradicated and also in malaria non endemic regions thus increasing the risk of reintroduction of malaria as well as spreading of malaria to new areas ( Martens & Hall, 2000). Other than transmitting Malaria, *Anopheles* mosquitoes have been indicated as a carriers of arboviruses including West Nile Virus and Japanese Encephalitis ( Thenmozhi *et al*., 2006), as well as viruses that cause O’nyong-nyong and Chikungunya fevers ( Vanlandingham *et al*., 2005). This study has indicated high diversity of *Anopheles* haplotypes in the Kisumu population, having detected *Anopheles gambiae* and *Anopheles funestus* ( Table 2). High diversity of *Anopheles* vector is a key feature for consideration in *Anopheles* management and has been associated with the rise in malaria transmission ( Loaiza *et al*., 2012). The low diversity of *Anopheles* species in Kilifi and Nairobi may be attributed to the Great Rift Valley, high-elevation mountains in western Kenya. The vast arid area east of the Great Rift Valley inhibits human settlement, thus restricting *Anopheles funestus* gene flow between coastal and western Kenya ( Lukindu *et al*., 2018). *Anopheles funestus* is closely associated with human dwellings and therefore plays an important role in the transmission of Malaria ( Kweka *et al*., 2013). *Anopheles gambiae* haplotypes in Kisumu were diverse, this is consistent with other studies that have reported a high genetic diversity of *Anopheles gambiae* in Kisumu Kenya ( Chen *et al*., 2004). Phylogenetic analysis ( Figure 3) and nucleotide diversity tests ( Table 4) showed no divergence between Kisumu *Anopheles gambiae* haplotype MK300234 with *Anopheles gambiae* MG753695.1, used as reference that was previously isolated in Uganda ( Lukindu *et al*., 2018). This indicates the presence of genetically identical *Anopheles gambiae* between Kenya and Uganda which could be attributed to cross-border migration across Lake Victoria. Therefore, this could suggest that, these species share the same ecological niche or ancestral divergence. *Anopheles gambiae (s.s.)* (formerly *Anopheles gambiae* S-form) is a main vector of malaria in sub-Saharan Africa, where 91% of an estimated 445,000 malaria deaths worldwide occurred in 2016 ( CDC - Malaria - About Malaria - Disease). Presence of both *Anopheles gambiae* and *Anopheles funestus* in Kisumu suggest that the area is still at high risk of malaria transmission. This study has identified a potentially new haplotype of *Anopheles* species MK300230 in Kilifi ( Figure 3). Through molecular techniques new haplotypes of *Anopheles* species are continually being identified; for instance, new species of *Anopheles nuneztovari* have been identified in Brazil ( Scarpassa *et al*., 2016).

*Culex pipiens* was distributed across Kilifi, Kisumu and Nairobi population while *Culex sitiens* was only identified in Kilifi population ( Table 1). *Culicidae* is a large and abundant group that occurs throughout temperate and tropical regions of the world, as well as the peri Arctic Circle ( Schäfer & Lundström, 2001). *Culex* mosquitos are an important vector of zoonotic infection Filariasis. Human filariasis infection is a major public health concern. Approximately 66% of those at risk of infection is in the South-East Asia Region and 33% in the African Region ( “WHO | World malaria report 2017,” 2018). Although *Culex pipiens* is ornithophilic it can also feed on humans and mammals ( Reisen *et al*., 1990; Reisen & Reeves, 1990) and thus capable to transmit West Nile Virus to Humans. *Culex pipiens* (Linnaeus) has been identified as the primary vector of West Nile virus ( Turell *et al*., 2000). Kenyan strain of *Culex pipiens* has been confirmed to be capable of transmitting West Nile virus and its circulation among humans in Kenya has been detected ( Lutomiah *et al*., 2011; Morrill *et al*., 1991). Therefore, the distribution of *Culex pipiens* across Kilifi, Nairobi and Kisumu could increase the risk of West Nile virus transmissions/outbreaks in most parts of Kenya. *Culex pipiens* haplotype MK300242 was identified in both Kilifi and Kisumu population ( Figure 4). This study reports distribution of identical mosquito vector species between populations. Phylogenetic analysis revealed *Culex pipiens* haplotype MK300242 from this study showed no divergence to the *Culex pipiens* sequences LC102132.1 from Portugal and KU380381.1, KU380372.1 from Nyanza Kenya ( Table 5). This study identified *Culex sitiens* in the Kilifi population only, *Culex sitiens* has been found to tolerate saline waters, in Oman it has been successfully isolated from brackish water ( Roberts, 1996). Consequently, parasites such as *Microsporidium, Amblyospora* have been isolated from *Culex sitiens* mosquito in Coastal Kenya ( Sabwa *et al*., 1984).

## Conclusion

This study has identified mosquito vectors that could spread arboviral pathogens distributed across Kilifi, Nairobi and Kisumu counties. The distribution varies in density where in some cases vector distribution is limited to particular areas which could be attributed to ecological and environmental variations.

## Data availability

### Underlying data

Culicidae cytochrome c oxidase subunit 1 (COI) gene, partial cds; mitochondrial. PopSet 1573759763: https://www.ncbi.nlm.nih.gov/popset/1573759763?report=genbank. Accession numbers MK300216 – MK300250
