# Supplementary material for: Diversity and Molecular Characterization of Mosquitoes (Diptera: Culicidae) in Selected Ecological Regions in Kenya
Source: F1000Res. 2019 Sep 24;8:262. Originally published 2019 Mar 6. [Version 2] doi: 10.12688/f1000research.18262.2 (PMC7255902; doi:10.12688/f1000research.18262.2)
Supplement: Peer review history

# Peer review: Reviewer response for version 2

**DOI:** 10.5256/f1000research.22664.r63163

**Published:** 2020-05-22

**Authors:** Kentaro Itokawa

**Affiliations:** 1 Pathogen Genomics Center, National Institute of Infectious Diseases, Tokyo, Japan

The study described in the manuscript authored by Makanda et al. describes the genetic diversity of mosquitoes captured in three regions in Kenya using the mitochondrial CO1 gene. They amplified a part of CO1 by PCR and sequenced the amplicon. Their analysis includes phylogenetic clustering of each CO1 haplotype with already registered sequence entries in Genbank and calculation of some basic population genetic parameters such as nucleotide diversity (Pi) and haplotype gene diversity (Hd). The main discovery and conclusions they have drawn out from the results were 

1. “A potentially novel *Anopheles* haplotype MK300230 was identified in Kilifi population.”
2.  “The first molecular characterization of *Aedes cumminsii* in Kenya”
3. “higher diversity of *Anopheles* haplotypes in the Kisumu population, having detected *Anopheles gambiae* and *Anopheles funestus*” 

 However, there are doubtful points at each finding they are claiming in this manuscript. Many of those problems seem to stem from species identification merely by sequence without leaving insect specimens for retrospective morphological assessment (they used the whole body of insects for DNA extraction, according to the manuscript). Because of this, it would be extremely difficult to address the concerns I will describe below.

**About the “A potentially novel *Anopheles* haplotype MK300230”**

   In the phylogenetic tree in Fig 3, the haplotype MK300230 locates outside of the known *Anopheles* group clade. This is very strange and interesting if that insect individual actually possessed morphological characters that look like *Anopheles*. Then I queried the sequence of MK300230 to GenBank database by blastn search and I found two notable hits, KY831299.1 (99% identical for 588 bp) and JN298693.1 (97% identical for 658 bp) both of which were registered as sequences obtained from some species of Tipuloidea. Unfortunately, MK300230 would not be a sequence for *Anopheles* or even not for a mosquito species. Probably, what the authors have actually captured was a kind of crane flies, though no clue to confirm it remains because the whole specimens have gone for DNA extraction.

**“The first molecular characterization of Aedes cumminsii in Kenya”**

   The claim that the insect represented by MK300225 belongs *Aedes cumminsii* is difficult to accept from the phylogenetic tree in Fig 2; there is only one reference sequence for *Aedes cumminsii *(MG242484). Even if MG242484 was the closest sequence entry to MK30025 in current database, it is a dangerous leap to conclude those two sequences are “haplotypes of same species”. With a similar reason, assigning MK300227 and MK300228 to *Aedes aegypti* in the Fig 2 and MK300247 to *Culex pipiens* in Fig 4 are also problematic because they are not clustered with reference sequences of each corresponding species at least in those figures.  

  Because of those issues with ambiguous species assignment of each haplotype sequences, I can not consider the calculated population genetic parameters as valid.

   Utilizing DNA sequence for taxonomical identification requires extreme care for the selection of reference sequences to be used. Sadly, the description of species associated with DNA sequences exiting in GenBank database is not always correct, but they are based on various levels of evidence. If one classifies his/her sequence merely based on similarity to a sequence that has a wrong species description and registered this new sequence with a wrong species assignment, this entry can be another source of another false finding by another researcher. At least, the authors should correct the ORGANISM section of MK300230 entry in GenBank which is now saying “Anopheles splendidus”.

  Preserving specimens is important to avoid many of those pitfalls. For mosquito, there are some useful method like NaOH crude extraction (e.g. Lars Rudbeck & Jørgen Dissing, 1998, BioTequniques 25 (4)), which enable preparing DNA template for PCR from a single leg with only cheap cost.The study described in the manuscript authored by Makanda et al described genetic diversity of mosquitoes captured in three regions in Kenya using mitochondrial CO1 gene. They amplified a part of CO1 by PCR and sequenced the amplicon. Their analysis includes phylogenetic clustering of each CO1 haplotype with already registered sequence entries in genbank and calculation of some basic population genetic parameters such as nucleotide diversity (Pi) and haplotype gene diversity (Hd). The main discovery and conclusions they have drawn out from the results were…

1.  “A potentially novel *Anopheles* haplotype MK300230 was identified in Kilifi population.”
2. “The first molecular characterization of *Aedes cumminsii* in Kenya”
3. “higher diversity of *Anopheles* haplotypes in the Kisumu population, having detected *Anopheles gambiae* and *Anopheles funestus*” 

However, there are doubtful points at each finding they are claiming in this manuscript. Many of those problems seem to stem on identification of species merely by sequence without leaving insect specimens for retrospective morphological assessment (they used whole body for DNA extraction according to the manuscript). Because of this, it would be extremely difficult to address the concerns I will describe below.

  

- About the “A potentially novel *Anopheles* haplotype MK300230”

In the phylogenetic tree in Fig 3, the haplotype MK300230 locates outside of the known *Anopheles* group clade. This is very strange and interesting if that insect individual actually possessed morphological characters which looks like *Anopheles*. Then I queried the sequence of MK300230 to genbank database by blastn search, I found two notable hits, KY831299.1 (99% identical for 588 bp) and JN298693.1 (97% identical for 658 bp) both of which were registered as sequences obtained from some species of Tipuloidea. Unfortunately, MK300230 would not be a sequence for *Anopheles* or even not for any mosquito species. Probably, what the authors have actually captured was a kind of crane flies, though no clue to confirm it remains if the whole specimens had been used for DNA extraction.

  

- “The first molecular characterization of *Aedes cumminsii* in Kenya”

The claim that the insect represented by MK300225 belong *Aedes cumminsii* is difficult to accept from the phylogenetic tree in Fig 2; there is only one reference sequence for *Aedes cumminsii *(MG242484). Even of MG242484 was the closest sequence entry to MK30025 in the current database, it is a dangerous leap to conclude those two sequences are “haplotypes of same species”. For a similar reason, assigning MK300227 and MK300228 to *Aedes aegypti* in Fig 2 and MK300247 to *Culex pipiens* in Fig 4 is also problematic because they are not clustered with reference sequences of each corresponding species at least in those figures.  

 Because of those problems,

 Utilizing DNA sequence for taxonomical identification requires extreme care for selection of reference sequences to be used. Sadly, the description of species associated with DNA sequences exiting in GenBank database is not always correct, but they are based on various levels of evidence. If one classifies his/her sequence merely based on similarity to a sequence that has a wrong species description and registered this new sequence with a wrong species assignment, this entry can be another source of another false finding by another researcher. At least, the authors should correct the ORGANISM section of MK300230 entry in GenBank which is now saying “ *Anopheles splendidus*” which is obviously incorrect.

  Preserving specimens is important to avoid many of those pitfalls. For mosquitoes, there are some useful methods like NaOH crude extraction (e.g. Lars Rudbeck & Jørgen Dissing, 1998, BioTequniques 25 (4)), which enable preparing DNA template for PCR from only a single leg with just a cheap cost.

**Suggestion**

  The author should revise the species assignment for each sequence more carefully and consider using ambiguous descriptions for less confident sequences (e.g. Aedes sp.).

Is the work clearly and accurately presented and does it cite the current literature?

Partly

If applicable, is the statistical analysis and its interpretation appropriate?

Partly

Are all the source data underlying the results available to ensure full reproducibility?

Yes

Is the study design appropriate and is the work technically sound?

Partly

Are the conclusions drawn adequately supported by the results?

Partly

Are sufficient details of methods and analysis provided to allow replication by others?

Yes

Reviewer Expertise:

Medical entomology; Genetics

I confirm that I have read this submission and believe that I have an appropriate level of expertise to confirm that it is of an acceptable scientific standard, however I have significant reservations, as outlined above.

# Peer review: Reviewer response for version 2

**DOI:** 10.5256/f1000research.22664.r54257

**Published:** 2019-10-03

**Authors:** Nicholas Johnson

**Affiliations:** 1 Animal and Plant Health Agency, Addlestone, UK

The authors have addressed the significant comments of the review. The Conclusions section is improved although replace "where's" with 'whereas'.

 No further comments.

Is the work clearly and accurately presented and does it cite the current literature?

Yes

If applicable, is the statistical analysis and its interpretation appropriate?

Not applicable

Are all the source data underlying the results available to ensure full reproducibility?

Partly

Is the study design appropriate and is the work technically sound?

Partly

Are the conclusions drawn adequately supported by the results?

Partly

Are sufficient details of methods and analysis provided to allow replication by others?

Yes

Reviewer Expertise:

Zoonotic viruses

I confirm that I have read this submission and believe that I have an appropriate level of expertise to confirm that it is of an acceptable scientific standard.

# Peer review: Reviewer response for version 1

**DOI:** 10.5256/f1000research.19974.r47313

**Published:** 2019-05-28

**Authors:** Laban Njoroge

**Affiliations:** 1 Invertebrate Zoology Section, The National Museums of Kenya, Nairobi, Kenya

Abstract and Introduction:

 There are a number of grammatical errors in the manuscript. The following are examples in the abstract:

 'In this study, we identified mosquito species across Kisumu, Kilifi and Nairobi Counties' - I believe mosquitoes were collected from only few places in the three counties and therefore should read were collected from and not across.

 There should be a word 'the' before consequent mortality in line 1 of the abstract.

 Commas are missing in some areas where they should be e.g. in the abstract.

 '.........Despite reports on increase of new and recurrent mosquito borne-disease outbreaks such as chikungunya, dengue fever and Rift valley fever in Kenya little is known about the genetic characteristics.' A comma after Kenya.

 'PCR was used to amplify and sequence the partial cytochrome oxidase subunit 1 (CO1) gene.' This sentence creates an impression that PCR was used for both amplificationa nd sequencing. I believe PCR was used to amplify the genes but sequencing was done using another method. This should be clarified.

 It is needless to repeat the word haplotypes three types.

 In the introduction: '.............pathogens causing diseases such as; malaria, lymphatic filariases, avian malaria' - That semi colon after such as should not be there.

 There is lack of standardization in writing names. e.g. disease names such as Chikungunya, Dengue are started with a capital letter in the main body but with small letters in the Keywords

 Some scientific names of some species are wrongly written e.g. Aedes cummnisii should be Aedes cumminsii. Culicine mosquitoes not Culcine mosquitoes in some areas and others mosquitos.

Methods:

 There aren't sufficient details of the methods to allow replication by others. For instance, there is no mention of where the Pyrethrum Spray Catches were done. Was in inside houses, Bus waiting lounges, garages?

 The reference for PSC collection method should clearly indicate that it is as used by Ndiath et al. 2011.

 I have rated the study design study as partly appropriate as being a study that targeted diversity, one sampling method that targets only indoor resting mosquitoes was not the best. There is probably a need to justify why only PSC was used and point one method as the reason for the low diversity collected. Again, it is not explained why bus stops in the three counties were preferred as sampling sites. If the idea was to see the contribution of transportation to the mixing of populations, then that didn't come out clearly. 

 'Only a few *Aedes aegypti* in Nairobi (Kinuthia *et al*., 2017)'. The authors do not tell us it is few of what. Few haplotypes or individuals?

 diversity and spread of *Aedes aegypti* has been associated with expansion on arboviral infection...........' - This statement needs to be rephrased. The word on can be replaced by of. Alternatively, it can be Diversity and spread of Ae. aegypti has been attributed to the increase in arboviral infections.

 'and was predominated in the Kilifi samples......' Should read  it was predominant in Kilifi samples.

 'This may contribute to the high susceptibility to dengue-outbreak reported in the region (Baba *et al' - *Should read this may contribute to their high susceptibility

 'The similarities in the genetic composition between the An. gambiae in Kenya and Uganda is most likely due to the proximity of the two countries to one another and the exchanges is more likely over land as opposed to across lake Victoria as claimed in the discussion.

 This study has indicated high diversity of *Anopheles* haplotypes in the Kisumu population' - I do not think two species only can be regarded as high diversity. Probably you should use the word higher in comparison with Nairobi and Kilifi.

 'The low diversity of *Anopheles* species in Kilifi and Nairobi may be attributed to the Great Rift Valley,' - there is an abundance of Anopheles especially in Kilifi (see Mwangangi et al 2012 which you have in the references). The problem is the choice of sampling method employed. PSC targets indoor resting mosquitoes only while the highest diversity are found outdoors

Conclusion:

 The conclusion is sounding a bit weak and it is more of a discussion than a conclusion. There isn't a strong conclusion about the findings on diversity and molecular characterization of mosquitoes encountered.

Is the work clearly and accurately presented and does it cite the current literature?

Yes

If applicable, is the statistical analysis and its interpretation appropriate?

I cannot comment. A qualified statistician is required.

Are all the source data underlying the results available to ensure full reproducibility?

Yes

Is the study design appropriate and is the work technically sound?

Partly

Are the conclusions drawn adequately supported by the results?

Partly

Are sufficient details of methods and analysis provided to allow replication by others?

Yes

Reviewer Expertise:

I am a mosquito taxonomist and ecologist. I am not an expert in sequencing and phylogenetic analyses and therefore that bit may require another expert.

I confirm that I have read this submission and believe that I have an appropriate level of expertise to confirm that it is of an acceptable scientific standard, however I have significant reservations, as outlined above.

## References

[1] : Abundance, diversity, and distribution of mosquito vectors in selected ecological regions of Kenya: public health implications. *J Vector Ecol*.2013;38(1) : 10.1111/j.1948-7134.2013.12019.x 134-42 10.1111/j.1948-7134.2013.12019.x 23701618

# Response

**Published:** 2019-09-11

**Authors:** Moni Makanda

**Affiliations:** JKUAT

First and foremost I would like to thank you for taking your time to review this article. Your views were most welcome and addressed accordingly. 

1. Grammatical errors were corrected throughout the article as highlighted.
2. Effect of transportation on mosquito diversity was not a focus in this study. This study looked in to the diversity of mosquitoes in the study sites being town areas.
3. The conclusion was strengthened as recommended.

# Peer review: Reviewer response for version 1

**DOI:** 10.5256/f1000research.19974.r47920

**Published:** 2019-05-13

**Authors:** Nicholas Johnson

**Affiliations:** 1 Animal and Plant Health Agency, Addlestone, UK

The article is a focused investigation of the haplotype variation observed in mosquito populations trapped at three locations in Kenya. The authors noted variation in all the 11 species reported including a number of novel observations. However, the authors do not include basic data on the actual distribution and species assemblage at the collection sites. By arbitrarily selecting 25 samples for extensive genetic analysis and ignoring the remaining samples that apparently included 894, 824 and 720 mosquitoes appears to be a fundamental omission. It is difficult to see how the authors can conclude that “The distribution varies in density” when the dataset has not been analysed. Whilst it may be beyond the resources of the team to molecularly type all 2,438 samples, without some attempt to include morphological identification of a significant proportion of these samples the manuscript is considerable diminished.

 The authors should check the capitalisation of pathogens throughout. As a general rule, names derived from a place are capitalised e.g. Rift Valley fever virus, whilst those that are not are in lower case e.g. yellow fever virus, malaria.

 The authors must revise the conclusions section to reflect the findings of the paper stating precisely what they have derived from their observations. At the moment the two sentences’ provide a revision of the manuscripts aim and a vague statement that is unsupported by the results.

 The reference for Morrill et al is incorrect. It should be Morrill et al., 1991, J Trop Med Hyg, 94, 166.

Is the work clearly and accurately presented and does it cite the current literature?

Yes

If applicable, is the statistical analysis and its interpretation appropriate?

Not applicable

Are all the source data underlying the results available to ensure full reproducibility?

Partly

Is the study design appropriate and is the work technically sound?

Partly

Are the conclusions drawn adequately supported by the results?

Partly

Are sufficient details of methods and analysis provided to allow replication by others?

Yes

Reviewer Expertise:

Zoonotic viruses

I confirm that I have read this submission and believe that I have an appropriate level of expertise to confirm that it is of an acceptable scientific standard, however I have significant reservations, as outlined above.

# Response

**Published:** 2019-09-11

**Authors:** Moni Makanda

**Affiliations:** JKUAT

First and foremost thank you for taking time to review the paper, your views were most welcomed and addressed.

1. It was beyond our financial capability to genetically analyse 2,438 samples. Earlier study by (Hale, 2012) support my study as adequate in phylogenetic analysis. However, morphological identification was done and further analysis by use of PCR-HRM. This data has been capture in my MSc. thesis, moreover a second publication on the same is underway. As this paper was focused on genetic diversity we focused on molecular analysis.
2. Names of pathogens throughout the article have been corrected based on the general rule.
3. The conclusion was strengthened as proposed.
4. Reference Morril et al. 1991 was revised as advised  
